# Syringe-push membrane absorption as a simple rapid method of urine preparation for clinical proteomics

**DOI:** 10.1186/s12014-015-9087-4

**Published:** 2015-06-06

**Authors:** Somchai Chutipongtanate, Channarong Changtong, Churat Weeraphan, Suradej Hongeng, Chantragan Srisomsap, Jisnuson Svasti

**Affiliations:** Central Laboratory, Department of Pediatrics, Faculty of Medicine Ramathibodi Hospital, Mahidol University, Bangkok, Thailand; Hematology and Oncology Unit, Department of Pediatrics, Faculty of Medicine Ramathibodi Hospital, Mahidol University, Bangkok, Thailand; Laboratory of Biochemistry, Chulabhorn Research Institute, Bangkok, Thailand; Applied Biological Sciences Program, Chulabhorn Graduate Institute, Bangkok, Thailand

**Keywords:** Comparison, Membrane absorption, Proteomics, Urine preparation

## Abstract

**Background:**

The analysis of urinary proteome might reveal biomarkers of clinical value. However, current methods of urine preparation for down-stream proteomic analysis are complicated, time-consuming, and/or expensive. This study aims to develop a robust, simple, inexpensive and readily accessible urine preparation method to facilitate clinical proteomic workflow.

**Result:**

Syringe-push membrane absorption (SPMA) was successfully developed by a combination of 5-ml medical syringe and protein-absorbable membrane. Comparing three membranes i.e., nitrocellulose, polyvinylidene difluoride (PVDF) and Whatman no.1, nitrocellulose combined with SPMA (nitrocellulose-SPMA) provided the greatest quality of proteome profile as demonstrated by 2-DE. The quality of the proteome profile and the performance of nitrocellulose-SPMA were systematically compared with three current methods of urine preparation (i.e., ultrafiltration, dialysis/lyophilization and precipitation). While different methods of urine preparation provided comparable proteome quality, nitrocellulose-SPMA had better working performance due to acceptable recovery yield, less workload, short working time, high accessibility and low unit cost. In addition, protein absorbed on nitrocellulose harvested from the SPMA procedure could be stored as a dried membrane at room temperature for at least 1-month without protein degradation or modification.

**Conclusions:**

SPMA is a simple rapid method of preparing urine for downstream proteomic analysis. Because of it is highly accessible and has long storage duration, this technique holds potential benefit for large-scale multi-center research and future development of clinical investigation based upon urinary proteomic analysis.

**Electronic supplementary material:**

The online version of this article (doi:10.1186/s12014-015-9087-4) contains supplementary material, which is available to authorized users.

## Background

Urine is an ideal source for clinical proteomic analysis due to non-invasive nature of sample collection [1, 2]. In addition, urine serves as a protein/peptide reservoir representing alterations in renal diseases [3, 4], non-renal localized conditions [5, 6] and systemic illnesses [7, 8]. Thus, the urinary proteome is a gateway to identify non-invasive biomarkers of clinical value. However, urine contains a relatively low amount of proteins, and is contaminated with a large amount of waste products (i.e., organic and non-organic molecules) which affect down-stream proteomic analysis [1, 2]. Therefore, good processing of urine samples is crucial for non-invasive biomarker discovery using the proteomic approach.

Commonly used methods of urine preparation for proteomic analysis include ultrafiltration [6, 9], dialysis with subsequent lyophilization [9, 10] and precipitation by organic solvents [9]. Those techniques provide a high quality urinary proteome profile. Nevertheless, some techniques are complicated, laborious and/or time-consuming, while others require specialized equipment and/or high-cost consumable supplies. A new method of urine preparation that is uncomplicated, robust, inexpensive and readily accessible would facilitate large-scale multi-center analysis and/or clinical investigations using urinary proteomic analysis in the future. This study therefore aimed to develop a simple rapid and reproducible method of urine preparation for clinical proteomics. Two-dimensional gel electrophoresis (2-DE), a mainstay approach of proteomics [11], was applied to evaluate the quality of proteome profile. The developed technique was systematically compared with the current methods of urine preparation in several aspects. The effect of storage duration on proteome stability was also investigated to highlight the usefulness of this technique in future large-scale research.

## Results and discussion

### Establishment of SPMA

The rationale of SPMA development was to devise a simple method of urine preparation that can be performed anywhere (even at the patient’s bedside in the primary hospitals) and is suitable for large-scale multi-center analysis, while preserving the best quality of urinary proteins for downstream proteomic analysis. Based on this conception, a novel technique was developed by combined use of a 5-ml medical syringe regularly used in clinical practice with membranes commonly used in clinical laboratory. The medical syringe was used as a pressure generator to push urine pass through the protein-absorbable membrane placed inside. Urinary proteins were absorbed onto the membrane surface and could be eluted later for proteomic analysis. Therefore, the “syringe-push” and “membrane absorption” were two core components of this development (Additional file 1: Figure S1).

**Fig. 1:**
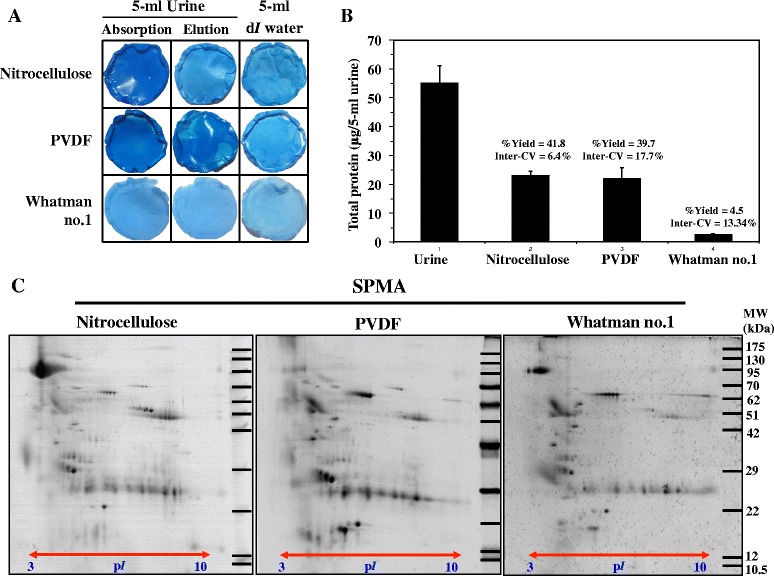
Silver Blue G-250 staining [12] showed deep blue staining of absorbed proteins on nitrocellulose and PVDF, but not Whatman no.1 membranes (**a**). Absorbed proteins were eluted by 2-D lysis buffer and measured by the Bradford protein assay for recovery yield and inter-CV (**b**). Eluted proteins were subsequently analyzed by 2-DE (50 μg/gel) with silver blue G-250 staining (**c**). All experiments were performed in triplicate

In order to select the most suitable membrane to combine with SPMA, three commonly used membranes available in clinical laboratories, namely nitrocellulose, PVDF and Whatman no.1, were tested and compared for protein adsorption capability in the SPMA procedure. Silver blue CBG-250 staining [12] was used to visualize the absorbed protein on each membrane. Figure 1a showed that nitrocellulose and PVDF, but not Whatman no.1, had deep blue staining after urine absorption as compared to faint blue staining after deionized (d*I*) water absorption (blank control). After elusion using 2-D lysis buffer, most proteins on nitrocellulose had been removed, whereas a significant amount of proteins on PVDF had still been retained. Note that incomplete elution of PVDF may affect recovery yield and reproducibility of protein isolation. This qualitative data supported the potential usage of nitrocellulose (more preferable) and PVDF (less preferable) in the SPMA technique. Next, urine protein isolates were quantitatively measured for recovery yield. Figure 1b showed that nitrocellulose and PVDF provided a comparable level of protein recovery yield (41.8 ± 2.7 % vs. 39.7 ± 7.0 %), whereas Whatman no.1 had very low yield (4.5 ± 0.6 %), consistent to previous qualitative result. The reproducibility of protein recovery yield of nitrocellulose (intra-CV of 6.4 %) was greater than PVDF and Whatman no.1 (intra-CV of 17.7 % and 13.3 %, respectively).

To evaluate compatibility of SPMA with down-stream proteomic analysis, urine proteins derived from three types of membrane were further analyzed by 2-DE (50 μg each gel). Figure 1c showed urinary proteome of nitrocellulose, PVDF and Whatman no.1, separated by 2-DE [11], stained with silver blue [12]. The samples from nitrocellulose provided more acidic proteins (p*I* range of 3–4) and had better quality of proteome profile than that of PVDF and Whatman no.1. For quantitative analysis, protein spot numbers were counted by ImageMaster 2D Platinum software. The results showed that nitrocellulose, PVDF and Whatman no.1 had 197 ± 15, 185 ± 16 and 163 ± 7 protein spots, respectively. According to recovery yield, reproducibility and quality of proteome profile, nitrocellulose was superior to PVDF and Whatman no.1, and was chosen for use with SPMA (nitrocellulose-SPMA) for subsequent evaluation.

### Systematic comparison of nitrocellulose-SPMA, ultrafiltration, dialysis/lyophilization and 75 % methanol precipitation

**Fig. 2:**
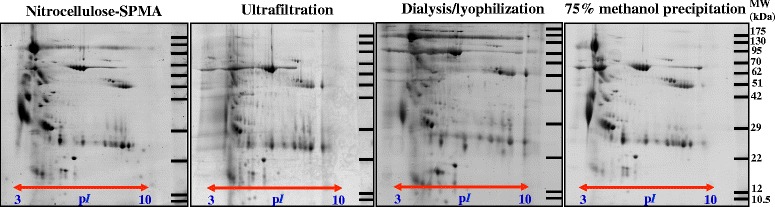
Urinary proteome profile of isolated proteins from nitrocellulose-SPMA, ultrafiltration, lyophilization, and 75 % methanol precipitation on 2-DE with silver blue G-250 staining (protein loading of 50 μg/gel). All experiments were performed in triplicate

Figure 2 showed urinary proteome profile of nitrocellulose-SPMA, compared to three current methods of urine preparation, including ultrafiltration, dialysis/lyophilization and 75 % methanol precipitation. Visually, all methods provided comparable quality with some differences in proteome pattern. The urinary proteome profile of nitrocellulose-SPMA was nearly identical to 75 % methanol precipitation, which is a preferred method of urine preparation for gel-based proteomics [9]. Quantitative analysis of protein spot numbers did not reveal significant differences between nitrocellulose-SPMA, ultrafiltration, dialysis/lyophilization, and 75 % methanol precipitation (197 ± 15, 196 ± 15, 192 ± 17 and 199 ± 17 spots, respectively) (*p* = 0.64) (Table 1). These results suggested that proteome coverage of urine protein isolated by nitrocellulose-SPMA was comparable to the standard methods of urine preparation. Nevertheless, some degree of variations among protocols could be expected, since different methods may possess different protein selectivity. To highlight the variation in protein selectivity, Western Blot analysis (Additional file 2: Supplementary materials and methods) was performed to detect four common urinary proteins, i.e., Tamm-Horsfall protein (THP), alpha-1-microglobulin (A1M), immunoglobulin gamma heavy chain (IgG HC), and immunoglobulin kappa chain (Ig Kappa) in the isolated proteins derived from nitrocellulose, PVDF and ultrafiltration (10 μg each lane) (Additional file 3: Figure S2). Based on four common urinary proteins, selectivity of nitrocellulose and ultrafiltration was nearly comparable, whereas PVDF provided the result with high degree of variation (which may be due to difference in protein selectivity or incomplete protein elution). This result again confirmed that nitrocellulose was more suitable than PVDF for SPMA technique.

**Table 1:** Systematic evaluation on performances of urine preparation methods

	Nitrocellulose-SPMA	Ultrafiltration	Dialysis/lyophilization	75 % methanol precipitation
Spot number mean ± SD (inter-CV)	197 ± 15 (7.6 %)	196 ± 15 (7.6 %)	192 ± 17 (8.8 %)	199 ± 17 (8.4 %)
%Yield mean ± SD (inter-CV)	41.9 ± 2.6 (6.4 %)	42.9 ± 0.6 (1.5 %)	62.1 ± 8.4 (13.6 %)	55.6 ± 7.0 (12.6 %)
Need of specialized/high-cost instrument	No	No	Yes (lyophilizer)	Yes (superspeed centifuge)
Working time (min)	15	35	>1440	65
Step of procedure	3	3	3	5
Unit cost (US$/5-ml urine)	0.3	6.3	0.3	1.8

*Abbreviation*: *inter-CV* inter-assay coefficient of variation

Performance of nitrocellulose-SPMA, ultrafiltration, dialysis/lyophilization, and 75 % methanol precipitation were compared and presented in Table 1. According to recovery yield, dialysis/lyophilization provided the highest recovery (62.1 ± 8.4 %) as compared to that of nitrocellulose-SPMA (41.9 ± 2.6 %), ultrafiltration (42.9 ± 0.6 %), and methanol precipitation (55.6 ± 7.0 %). This result was not surprising because dialysis/lyophilization usually provides the highest recovery yield [9]. Although nitrocellulose-SPMA had lower recovery yield compared to dialysis/lyophilization and 75 % methanol precipitation, the protein recovered was comparable to ultrafiltration which is adequate for proteomic analysis. To evaluate the accessibility of urine preparation protocol, the need for specialized/high cost instrumentation also needs to be considered. Nitrocellulose-SPMA and ultrafiltration do not need specialized/high cost instrument, whereas dialysis/lyophilization and 75 % methanol precipitation required a lyophilizer and superspeed centrifuge, respectively. This would be advantageous in large-scale proteomic analysis using urine specimens initially prepared from less accessible regions. In clinical investigation, fewer steps in the protocol procedure and less working time decrease workload. Of the four methods, only 75 % methanol precipitation requires a 5-step of procedure, while nitrocellulose-SPMA, ultrafiltration, dialysis/lyophilization had 3-step procedures (detailed in Materials and Methods section). Thus, 75 % methanol precipitation was more complicated to perform than the others. In our experience, urine preparation by precipitation techniques also requires careful training to obtain a reliable and reproducible 2-DE result. The approximate working times of nitrocellulose-SPMA, ultrafiltration, dialysis/lyophilization and 75 % methanol precipitation were shown in Table 1 (detailed in Materials and Methods section). Clearly, nitrocellulose-SPMA had the least working time as compared to the others. In terms of unit cost, nitrocellulose-SPMA and dialysis/lyophilization cost only 0.3 US$ per 5-ml urine preparation, whereas ultrafiltration (6.3 US$) and 75 % methanol precipitation (1.8 US$) had higher cost. Taken all these factors into account, nitrocellulose-SPMA had better working performance and was also cost-effective as compared to the other methods.

### Storage duration of urine protein-absorbed nitrocellulose membrane

**Fig. 3:**
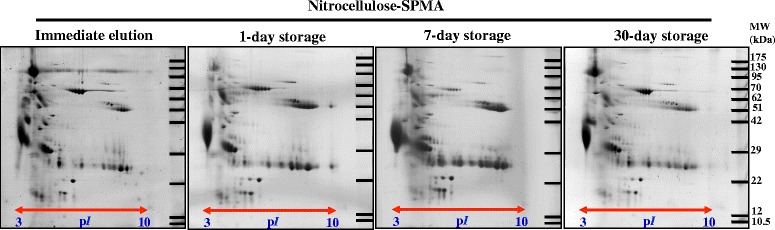
Storage duration of urinary proteins absorbed on the nitrocellulose membrane after SPMA procedure. After urine protein absorption, the membrane was air dried and kept at room temperature until elution at 1-day, 1-week and 1-month. Immediate elution served as control condition. 2-DE was performed to assess protein degradation and/or modification after long storage. No change of urinary proteome was observed up to 1-month

To emphasize the usefulness of nitrocellulose-SPMA in clinical proteomics and large-scale multi-center analysis, storage duration of the dried protein-absorbed membrane was investigated. Membrane containing adsorbed urinary protein was prepared by nitrocellulose-SPMA and then air dried before elution at the desired time points. 2-DE was chosen for this evaluation due to its high sensitivity to protein degradation (which can be observed as vertical smearing) and protein modification (which can be observed as horizontal shifting) [11]. Figure 3 shows the 2-DE of urinary proteins eluted from nitrocellulose-SPMA with different storage times under room temperature. Interestingly, there were no difference in proteome profile among four conditions including immediate elution, 1-day storage, 1-week storage and 1-month storage. Neither vertical smear nor horizontal shift of protein spots was observed. This result suggested that urinary protein absorbed to the membrane may be kept for up to 1 month for analytical purposes.

### Potential applications of SPMA technique on large-scale analysis and future development of clinical investigation based upon urinary proteomic technologies

Urinary proteomics is the promising tool for biomarker discovery of clinical value, particularly when performed in a large-scale fashion. However, a limitation for large-scale analysis is sample preparation (due to complicated protocol, lack of equipment and/or inadequate funding, especially in developing countries) and sample shipment as cooled/frozen specimens. The newly develop SPMA overcomes those limitations. The simplicity and accessibility of SPMA can overcome bottlenecks due to difficult sample preparation and handling in urinary proteomics. Because of its robustness and low unit cost, SPMA is therefore compatible with handling a large number of urine specimens in a clinical setting. Long storage stability of the dried urine protein-absorbed membrane (up to 1-month at room temperature) allows specimen collection from rural areas for large-scale research and/or clinical investigation based upon urinary proteomic analysis (Additional file 4: Figure S3). This also overcomes problems due to shipping of urine specimens (i.e., protein stability, apparatus for packaging and shipping expense due to liquid storage and weight). All of these highlight the cost-effectiveness of nitrocellulose-SPMA in clinical proteomics.

Recently, a PVDF-based urine protein absorption using vacuum suction filter bottle system was described by Jia L, et al., in order to preserve urine proteins for large-scale prospective studies [13]. Reproducibility of urinary protein isolation and protein stability after 18-day storage at various temperatures (i.e., room temperature, 4 °C, −20 °C and −80 °C) were demonstrated using SDS-PAGE analysis. Unfortunately, proteome coverage and protein modification had not been evaluated due to the limitation of SDS-PAGE analysis. By changing PVDF to nitrocellulose, the same group of investigators showed that urinary proteins derived from nitrocellulose membrane absorption had the same degree of reproducibility and proteome coverage as those of acetone precipitation by LC-MS/MS analysis [14]. However, the advantages of membrane absorption technique over the other conventional methods (e.g., ultrafiltration or dialysis/lyophilization) have remained unclear. Also, a rationale of membrane selection (PVDF vs. nitrocellulose) has never been described.

Our study has provided additional information to the previous works. The evaluation was performed in a systematic manner. The compact design makes SPMA more economical and more accessible than the vacuum suction filter bottle system. Nitrocellulose was superior to PVDF and Whatman no.1 in term of yield, reproducibility and proteome quality of the isolated urinary proteins, making the rationale of membrane selection for this procedure. Importantly, nitrocellulose and PVDF are not replaceable because of different proteome profile (Fig. 1c). For large-scale urinary protein collection based on membrane absorption techniques (either SPMA or vacuum suction filter bottle system), making a right decision on membrane selection is crucial. Advantages of SPMA over the conventional methods including ultrafiltration, dialysis/lyophilization and 75 % methanol precipitation were clearly demonstrated in the aspects of working performance and cost-effectiveness. One-month storage time of urine protein-absorbed membrane at room temperature, without protein degradation and/or modification, was an additional benefit to this technique. By step-wise evaluation, nitrocellulose-SPMA was highlighted as a method of choice of urine preparation for clinical proteomic analysis, especially by the gel-based approach.

In conclusion, nitrocellulose-SPMA described here is a robust, simple, inexpensive and readily accessible method of urine preparation for clinical proteomics. Nitrocellulose-SPMA is not only has benefits for research purposes, but also for clinical investigations based upon proteomic technologies, thus supporting the future development of clinical proteomics.

## Materials and methods

### Urine collection

The international urine collection protocol created as a result of joint consensus of European Kidney and Urine Proteomics (EuroKUP) [15] and the Human Kidney and Urine Proteome Project [16] (http://eurokup.org) was followed. Mid-stream of 2nd morning urine from six normal healthy individuals (three males; age of 34.3 ± 6.4 years and three females; age of 29.0 ± 4.3 years) were collected with 1 mM sodium azide and kept at 4 °C. The collected urine was pretreated by centrifugation at 1000 × g for 10 min to remove cells and debris. Pooled urine was then generated from 100 ml urine per individual, stored aliquots of 10 ml at −20 °C, and used throughout this study. This study was approved by the Ethical Clearance Committee on Human Right Related to Research involving Human Subjects, Faculty of Medicine Ramathibodi Hospital, Mahidol University (Protocol ID 01-58-06).

### Establishment of SPMA technique

A schematic diagram represented SPMA was showed in Additional file 1: Figure S1. Three commonly available membranes including nitrocellulose (Bio-Rad Laboratories Inc., Hercules, CA), polyvinylidene difluoride (PVDF) and Whatman no.1 filter membrane (Whatman no.1) were selected for this study. The membrane was cut into a disc-shaped membrane with an approximate diameter of 1.2 cm. The disc-shaped membrane was carefully installed at the bottom of a 5 ml medical syringe (Terumo Medical Corp., Somerset, NJ). Complete sealing of the syringe outlet by the disc-shaped membrane is necessary to maximize the yield of protein absorption. Optionally, Whatman ashless grade 40 filter paper (a diameter of 1.2 cm) was inserted before installation of the membrane to prevent leakage. For urinary protein absorption, 5-ml urine was filled into a membrane-filled syringe and then pushed through the membrane using the plunger at an approximate flow rate of 1 ml/min. Positive pressure generated by pushing the syringe is crucial to absorb urinary proteins onto the membrane. To eliminate salt and waste product contamination, 5-ml d*I* water was push through the protein-absorbed membrane at the similar flow rate. The protein-absorbed membrane was harvested and incubated with 2-D lysis buffer (7 M urea, 2 M thiourea, 4 % CHAPS (3-[(3-Cholamidopropyl) dimethylammonio]-1-propanesulfonate), 2 % (v/v) IPG pH 3–10, 120 mM dithiothreital (DTT) and 40 mM Tris (1,3-Propanediol, 2-amino-2-(hydroxymethyl)) in an Eppendorf tube for 5-min to elute urinary proteins. For long-term storage, the harvested membrane was air dried and kept at room temperature in a clean plastic bag until use. Protein concentration was measured using Bradford protein assay.

### Ultrafiltration

To obtain the best proteome coverage by ultrafiltration, 3-kDa MWCO microsep™ advanced centrifugal filter (Pall Life Science, Ann Arbor, MI) was used. Ultrafiltration was performed as described previously [6, 9]. Briefly, 5-ml urine was spun at 12,000 × g using an ultrafiltration column at 4 °C until approximately 1/30 of initial volume remained. Ten volumes of 90 mM Tris–HCl, pH 7.4 was used as a changing buffer in order to get rid of salt contamination. The concentrated urinary protein was harvested and kept at −20 °C until use. Protein concentration was measured using the Bradford protein assay.

### Dialysis/lyophilization

Urine preparation by dialysis and lyophilization was performed as described previously [9, 10]. Briefly, 5-ml urine was filled in a dialysis bag with a molecular weight cut off (MWCO) of 3–5 kDa and then dialyzed against d*I* water at 4 °C overnight with a total dilution factor of 16,000. The dialyzed urine was then lyophilized using a freeze dryer for about 6-h or until completely dry. The lyophilized urinary protein was then resuspended in 2-D lysis buffer and kept −20 °C until used. Protein concentration was measured using the Bradford protein assay.

### 75 % methanol precipitation

Various organic solvents may be used to precipitate urinary proteins but no single solvent is perfect [9]. However, previous study showed that 75 % methanol precipitation provided a good quality proteome profile, high recovery yield and was easy to handle, compared to other solvents [9]. Therefore, 75 % methanol precipitation was selected as a representative precipitation technique and was performed as described previously [9]. Briefly, methanol and the pooled urine were pre-cooled at 4 °C for 30 min before adding methanol into the urine to the final concentration of 75 % v/v. The solution was mixed and incubated at 4 °C for 10 min to allow protein precipitation. The precipitant was isolated by superspeed centrifugation at 12,000 g for 5 min. The supernatant was carefully discarded and the pellet was air-dry under room temperature for 15 min and then resuspended using 2-D lysis buffer. Protein concentration was measured using the Bradford protein assay.

### 2-DE and staining

Fifty micrograms of extracted protein was mixed with rehydration buffer (7 M urea, 2 M thiourea, 4 % CHAPS, 0.5 % (v/v) IPG buffer pH 3–10, 60 mM DTT and 40 mM Tris) and rehydrated into a 7-cm IPG strip (pH 3–10) for 10–15 h at room temperature. The first dimension separation or isoelectric focusing (IEF) was performed by the Ettan IPGphor III IEF System (GE Healthcare, Little Chalfont, UK) at 20 °C using a stepwise voltage increase to reach 9000 Vh. The focused IPG strip was equilibrated with an equilibration buffer (6 M urea, 130 mM DTT, 112 mM Tris–HCl pH 8.8, 4 % SDS, 30 % glycerol and 0.002 % bromophenol blue) for 15 min, followed by a second equilibration for 15 min in the same solution containing 135 mM iodoacetamide instead of DTT. The equilibrated strip was transferred to the top of a 12.5 % polyacrylamide gel and then second dimensional separation was performed using SE260 mini-Vertical Electrophoresis Unit at 150 V for approximately 2 h. Protein spots were visualized by silver blue CBG-250 staining [12]. The stained gel was scanned by Ettan DIGE Imager (GE Healthcare). Numbers of protein spot were detected using ImageMaster 2D-Platinum software version 6.0 (GE Healthcare).

### Comparisons among urine preparation methods

#### Quality of proteome profile

Quality of proteome profile was judged by visualized mapping of the pattern and resolution of protein spots on 2-DE [11], and quantitatively evaluated by the numbers of protein spots detected by ImageMaster 2D-Platinum software. Data was presented as mean ± SD and inter-assay coefficient of variation (inter-CV) of three independent experiments.

#### Recovery yield

Total protein amount of pooled urine and the isolated protein (based on a total volume of 5-ml urine each) were measured by the Bradford assay. Recovery yield was calculated as followed;

$$ \%\;\mathrm{Yield}=\frac{\mathrm{Total}\ \mathrm{protein}\ \mathrm{amount}\ \mathrm{of}\ \mathrm{the}\ \mathrm{isolated}\ \mathrm{protein}\ \left(\mathrm{mg}\right)\times 100}{\mathrm{Total}\ \mathrm{protein}\ \mathrm{amount}\ \mathrm{of}\ \mathrm{the}\ \mathrm{pooled}\ \mathrm{urine}\ \left(\mathrm{mg}\right)} $$

Where, [total protein amount of the isolated protein (mg) = protein concentration of the eluate (mg/ml) × the volume of the elute (ml)]. Data was presented as mean ± SD and inter-CV of three independent experiments.

#### Steps in procedure and working time

Steps in each procedure and approximated working time were estimated based upon the manipulation of 5-ml urine as follows: 3 steps for SPMA (5-min for membrane absorption, 5-min for washing, and 5-min for elution), 3 steps for ultrafiltration (15-min for protein concentration, 15-min for desalting and 5-min for harvesting), 3 steps for dialysis/lyophilization (960-min for dialysis, 360-min or more for lyophilization, 5-min for solubilization) and 5 steps for precipitation (30-min for pre-cooling, 10-min for precipitation, 5-min for superspeed centrifugation, 15-min for air drying, and 5-min for solubilization).

#### Accessibility and unit cost

Accessibility of urine preparation method was evaluated by the need of specialized/high cost instruments (defined by cost greater than 12,500 US$). Based on this, two methods, namely dialysis/lyophilization and precipitation (which require a freeze dryer and a superspeed centrifuge, respectively) were categorized as being low accessibility techniques. Unit cost was approximated by the cost of consumable materials used for 5-ml urine manipulation (US$/5-ml urine).

### Statistical analysis

Statistical analysis was performed with SPSS version 11.5 (SPSS, Inc., Chicago, IL). Data were analyzed by unpaired *t*-test or ANOVA to determine the difference between groups. All experiments were performed in triplicate and presented as mean ± SD. *P*-value < 0.05 is considered as statistical significance.

## Additional files

Additional file 1: Figure S1. Schematic diagram representing the SPMA procedure. A 5-ml medical syringe was prefilled with a disc-shape protein absorbable membrane (A). Five-ml of urine was pushed through the membrane using a plunger to enhance protein absorption (B and C). Urinary proteins were eluted from the harvested membrane using 2-D lysis buffer and prepared for subsequent proteomic analysis (D). SPMA; syringe-push membrane absorption.

Additional file 2: **Supplementary materials and methods.**

Additional file 3: Figure S2. Western blotting using specific antibodies against 4 abundant urinary proteins, i.e., Tamm-Horsfall protein (THP), immunoglobulin gamma heavy chain (IgG HC), alpha-1-microglobulin (A1M) and immunoglobulin kappa chain (Ig Kappa) were performed to evaluate protein selectivity of nitrocellulose and PVDF. Urinary protein derived from ultrafiltration was served as the control condition. Equal amount of 10 μg protein was loaded in each lane. The data shown was representative of triplicate experiments.

Additional file 4: Figure S3. Proposed usefulness of nitrocellulose-SPMA in large-scale multicenter proteome research and/or clinical investigation based upon urinary proteomic analysis. A. Urine protein specimens are prepared by nitrocellulose-SPMA at a primary hospital and sent as dried membranes via mail to the central hospital and/or the research institute. B. Proteins are eluted and submitted to proteomic analysis. C. For research purposes, relevant pathogenic mechanisms and/or biomarkers of clinical value can be identified by large-scale analysis. D. Later, when clinical investigation based on proteomic technologies are complete, the results are sent back to primary hospital for further patient care.
