# Supplementary figures and images for: Syringe-push membrane absorption as a simple rapid method of urine preparation for clinical proteomics
Source: Clin Proteomics. 2015 Jun 6;12(1):15. doi: 10.1186/s12014-015-9087-4 (PMC4464716; doi:10.1186/s12014-015-9087-4)

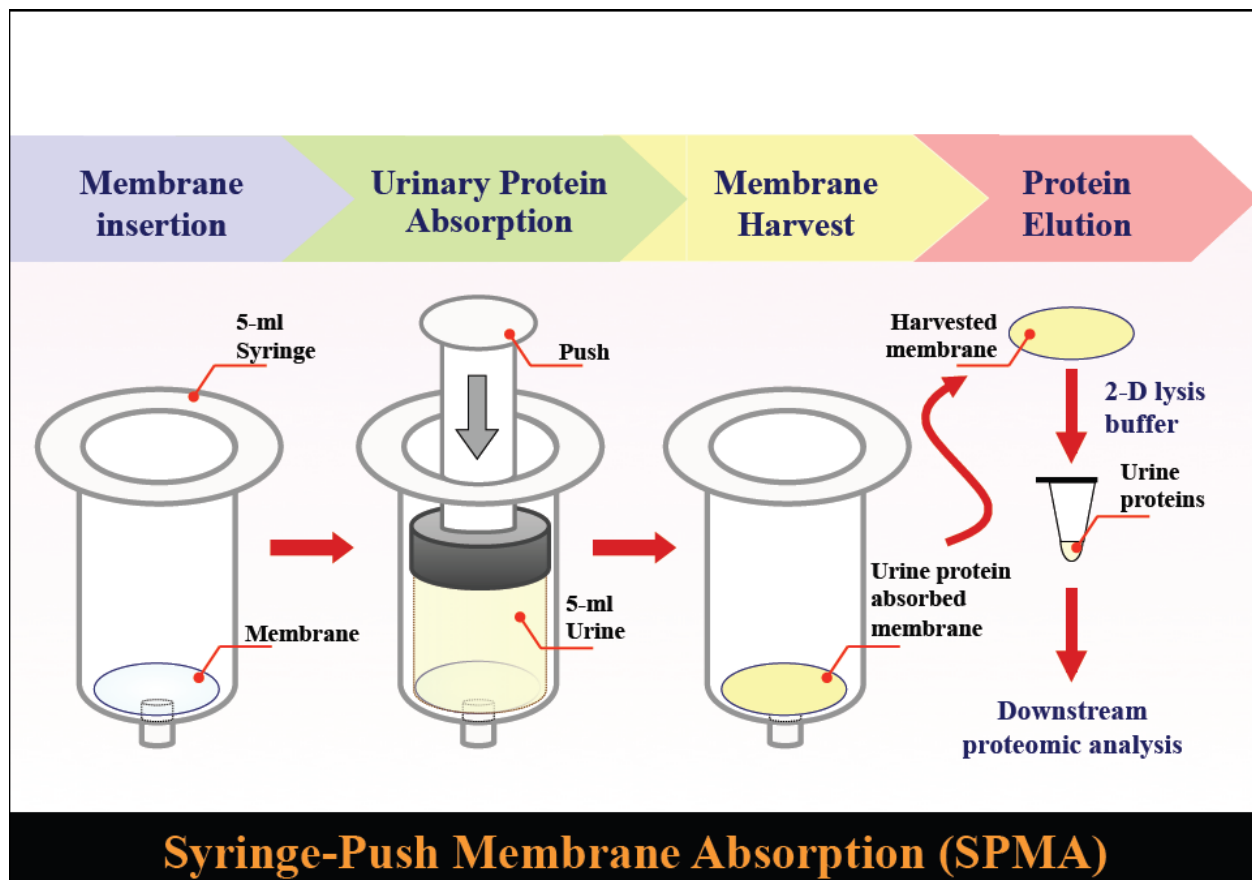

Supplementary figure 1

Supplement: Supplementary file 1 — Schematic diagram representing the SPMA procedure. A 5-ml medical syringe was prefilled with a disc-shape protein absorbable membrane (A). Five-ml of urine was pushed through the membrane using a plunger to enhance protein absorption (B and C). Urinary proteins were eluted from the harvested membrane using 2-D lysis buffer and prepared for subsequent proteomic analysis (D). SPMA; syringe-push membrane absorption. [file 12014_2015_9087_MOESM1_ESM.pdf]

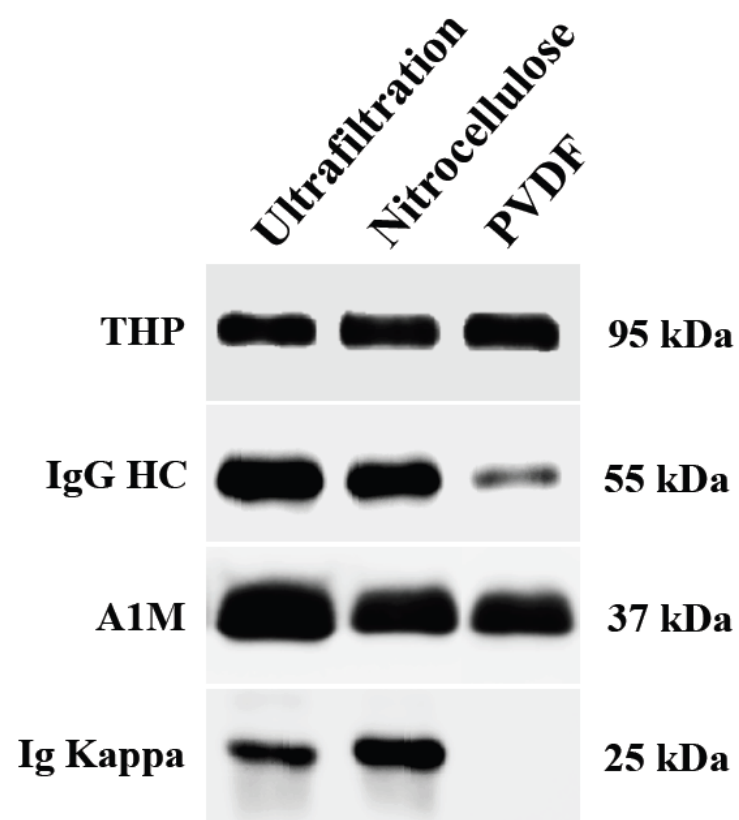

Supplementary figure 2

Supplement: Supplementary file 3 — Western blotting using specific antibodies against 4 abundant urinary proteins, i.e., Tamm-Horsfall protein (THP), immunoglobulin gamma heavy chain (IgG HC), alpha-1-microglobulin (A1M) and immunoglobulin kappa chain (Ig Kappa) were performed to evaluate protein selectivity of nitrocellulose and PVDF. Urinary protein derived from ultrafiltration was served as the control condition. Equal amount of 10 μg protein was loaded in each lane. The data shown was representative of triplicate experiments. [file 12014_2015_9087_MOESM3_ESM.pdf]

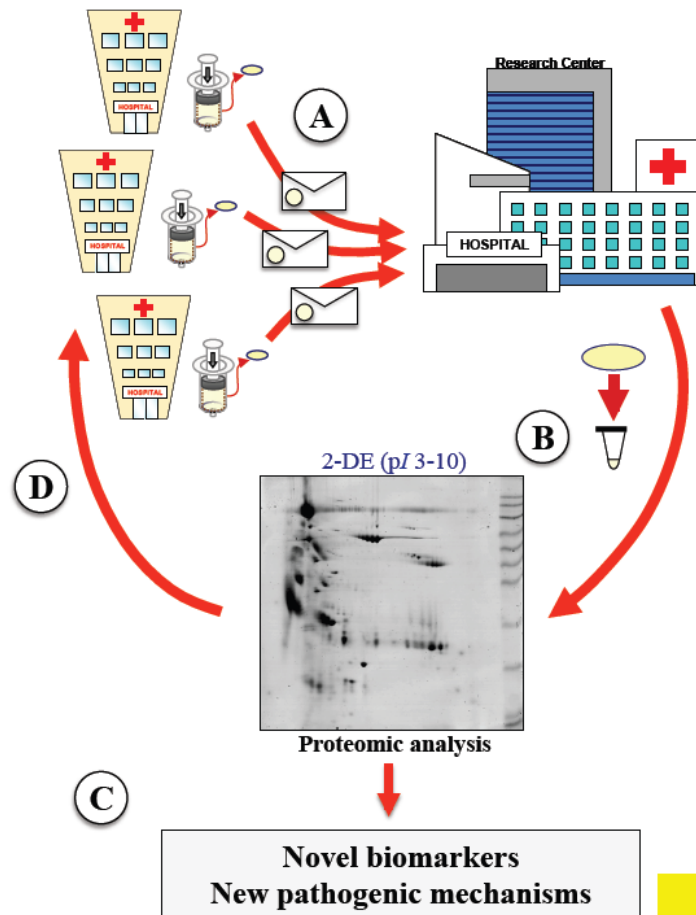

Supplementary figure 3

Supplement: Supplementary file 4 — Proposed usefulness of nitrocellulose-SPMA in large-scale multicenter proteome research and/or clinical investigation based upon urinary proteomic analysis. A. Urine protein specimens are prepared by nitrocellulose-SPMA at a primary hospital and sent as dried membranes via mail to the central hospital and/or the research institute. B. Proteins are eluted and submitted to proteomic analysis. C. For research purposes, relevant pathogenic mechanisms and/or biomarkers of clinical value can be identified by large-scale analysis. D. Later, when clinical investigation based on proteomic technologies are complete, the results are sent back to primary hospital for further patient care. [file 12014_2015_9087_MOESM4_ESM.pdf]
