# Supplementary material for: Syringe-push membrane absorption as a simple rapid method of urine preparation for clinical proteomics
Source: Clin Proteomics. 2015 Jun 6;12(1):15. doi: 10.1186/s12014-015-9087-4 (PMC4464716; doi:10.1186/s12014-015-9087-4)
Supplement: Supplementary file 2 — Supplementary materials and methods. [file 12014_2015_9087_MOESM2_ESM.pdf]

SUPPLEMENTARY MATERIALS AND METHODS

Western Blotting

Proteins (10 μg/lane) were resolved in 12.5% SDS-PAGE at a constant 10 mA/gel for 3 h. The separated proteins were electrotransferred onto PVDF membranes (Immobilion-P; Millipore, MA, USA), following by probing membranes using antibodies against Tamm-Horsfall urinary glycoprotein (1:500) (ab167678; Abcam Inc., Cambridge, MA), or human alpha-1-microglobulin (1:1000) (ab129059; Abcam Inc.) at 4°C overnight. Membranes were washed and then incubated with corresponding secondary antibody conjugated with HRP (1:1000) (DakoCytomation, Denmark) at room temperature for 1 h. For antibody against human IgG conjugated with HRP (1:5000) (P0214; DakoCytomation) and Ig kappa chain conjugated with HRP (1:1000) (P 0212; DakoCytomation), membranes were probed at room temperature for 1 h. Membranes were incubated with enhanced chemiluminescence (ECL) (GE Healthcare), followed by detection with ImageQuant™ LAS 4000 (GE Healthcare). The data shown was representative of triplicate experiments.
